# Plasma Exosome Profile in ST-Elevation Myocardial Infarction Patients with and without Out-of-Hospital Cardiac Arrest

**DOI:** 10.3390/ijms22158065

**Published:** 2021-07-28

**Authors:** Marta Zarà, Jeness Campodonico, Nicola Cosentino, Maria Luisa Biondi, Patrizia Amadio, Gloria Milanesi, Emilio Assanelli, Silvia Cerri, Marco Biggiogera, Leonardo Sandrini, Calogero Claudio Tedesco, Fabrizio Veglia, Daniela Trabattoni, Fabio Blandini, Elena Tremoli, Giancarlo Marenzi, Silvia S. Barbieri

**Affiliations:** 1 Centro Cardiologico Monzino IRCCS, 20138 Milan, Italy; marta.zara@ccfm.it (M.Z.); jeness.campodonico@ccfm.it (J.C.); nicola.cosentino@ccfm.it (N.C.); maria.biondi@ccfm.it (M.L.B.); patrizia.amadio@ccfm.it (P.A.); emilio.assanelli@ccfm.it (E.A.); leonardo.sandrini@ccfm.it (L.S.); calogero.tedesco@ccfm.it (C.C.T.); fabrizio.veglia@ccfm.it (F.V.); daniela.trabattoni@ccfm.it (D.T.); elena.tremoli@ccfm.it (E.T.); giancarlo.marenzi@ccfm.it (G.M.); 2 Department of Clinical Sciences and Community Health, Cardiovascular Section, University of Milan, 20122 Milan, Italy; 3 Department of Biology and Biotechnology, University of Pavia, “L. Spallanzani”, 27100 Pavia, Italy; gloria.milanesi@unipv.it (G.M.); marco.biggiogera@unipv.it (M.B.); 4 Mondino Foundation IRCCS, 27100 Pavia, Italy; silvia.cerri@mondino.it (S.C.); fabio.blandini@mondino.it (F.B.); 5 Department of Brain and Behavioural Sciences, University of Pavia, 27100 Pavia, Italy

**Keywords:** exosomes, ST-elevation myocardial infarction, resuscitated out-of-hospital cardiac arrest, platelets, brain-associated marker

## Abstract

The identification of new biomarkers allowing an early and more accurate characterization of patients with ST-segment elevation myocardial infarction (STEMI) is still needed, and exosomes represent an attractive diagnostic tool in this context. However, the characterization of their protein cargo in relation to cardiovascular clinical manifestation is still lacking. To this end, 35 STEMI patients (17 experiencing resuscitated out-of-hospital cardiac arrest (OHCA-STEMI) and 18 uncomplicated) and 32 patients with chronic coronary syndrome (CCS) were enrolled. Plasma exosomes were characterized by the nanoparticle tracking analysis and Western blotting. Exosomes from STEMI patients displayed a higher concentration and size and a greater expression of platelet (GPIIb) and vascular endothelial (VE-cadherin) markers, but a similar amount of cardiac troponin compared to CCS. In addition, a difference in exosome expression of acute-phase proteins (ceruloplasmin, transthyretin and fibronectin) between STEMI and CCS patients was found. GPIIb and brain-associated marker PLP1 accurately discriminated between OHCA and uncomplicated STEMI. In conclusion, the exosome profile of STEMI patients has peculiar features that differentiate it from that of CCS patients, reflecting the pathophysiological mechanisms involved in STEMI. Additionally, the exosome expression of brain- and platelet-specific markers might allow the identification of patients experiencing ischemic brain injury in STEMI.

## 1. Introduction

ST-segment elevation myocardial infarction (STEMI) is a life-threatening and time-dependent emergency that must be diagnosed and treated promptly by pharmacologic and/or mechanical myocardial reperfusion [1]. The outcome of patients with STEMI has significantly improved over the last decades due to progress in drug therapy and interventional techniques [2]. Nevertheless, as STEMI patients still represent a heterogeneous population in terms of in-hospital mortality risk and recurrent cardiovascular events [3], there is an impelling need for the identification of new tools that allow early and more accurate patient characterization.

Recent attention has been given to exosomes as attractive diagnostic and prognostic tools in several clinical conditions [4], including cardiovascular diseases [5,6]. Exosomes are small extracellular vesicles (30–150 nm in diameter) originating from multivesicular bodies, actively released by different cell types and detectable in several body fluids, including plasma. Exosomes play a central role in mediating both local (paracrine) and distant (endocrine) intercellular communication, and by transferring their bioactive cargo (DNA, chemokines, messenger RNAs, microRNAs and proteins), they modulate the behavior of recipient cells [7,8,9]. The growing knowledge about exosomes has highlighted their involvement in nearly all aspects of physiology and pathology [4]. As exosomal release can be triggered by several stimuli associated with cellular stress [9,10,11,12,13,14], exosome secretion from platelets, vascular endothelial cells and brain cells, may be modulated in several pathological conditions, including myocardial infarction and stroke. Moreover, the content of exosomes, representing a “fingerprint” of the releasing cell and its metabolic status, might be a rich source of biomarkers for various disease states [4,9,15].

In the clinical setting of acute myocardial infarction, exosome-associated microRNAs have been widely investigated [5,16,17]. Conversely, in this pathological condition, the characterization of exosome protein cargo is still lacking. To start filling this gap, in this study, we characterized the signature of plasma exosomes isolated from STEMI patients and compared it to that of patients with chronic coronary syndrome (CCS) considered as the control group. Moreover, since exosomes released from brain cells are able to cross the blood–brain barrier (BBB) [18,19,20], we hypothesized that their investigation might be helpful to unravel the cross-talk between heart and brain in STEMI patients with out-of-hospital resuscitated cardiac arrest. Thus, we analyzed the exosome protein cargo of STEMI patients with and without cardiac arrest before hospital presentation. Specifically, in this study, we focused on a panel of proteins already known to be carried by plasma exosome [21], possibly reflecting their cellular origin and/or well-known major pathophysiological mechanisms involved in the acute phase of STEMI [22,23,24,25,26,27,28,29].

## 2. Results

### 2.1. Patient Characteristics

Thirty-five STEMI patients were enrolled in the study. Of them, 17 were OHCA-STEMI whereas the remaining 18 were uncomplicated STEMI. Moreover, 32 CCS patients were included as controls. Clinical characteristics and laboratory data at hospital admission of the study population are reported in Table 1. 

### 2.2. Exosome Characterization in Uncomplicated STEMI Versus CCS

**Figure 1:**
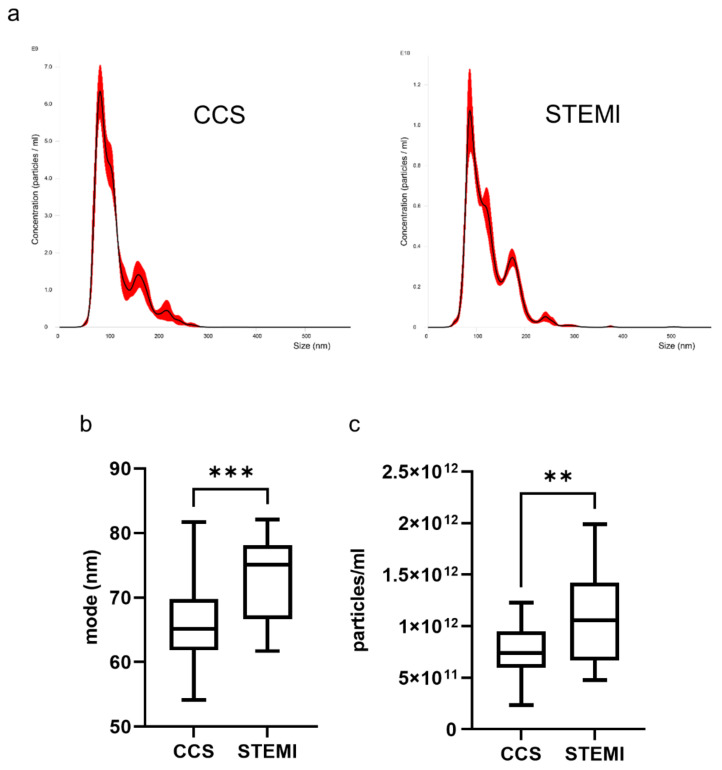
Exosome concentration and dimension are different between CCS and STEMI patients. Exosomes were isolated from plasma of CCS (N = 32) and STEMI (N = 18) patients and analyzed by NTA. (**a**) Representative NTA traces of exosomes isolated from plasma of CCS and STEMI patients. (**b**,**c**) Analysis of exosome dimension (mode) (**b**) and concentration (number of particles/mL) (**c**) as assessed by NTA. For each box plot, the center line illustrates the median and box limits indicate the 25th and 75th percentiles. ** *p* < 0.01, *** *p* < 0.001.

Nanoparticle tracking analysis showed that uncomplicated STEMI patients had larger and more numerous plasma exosomes than CCS patients (Figure 1). 

**Figure 2:**
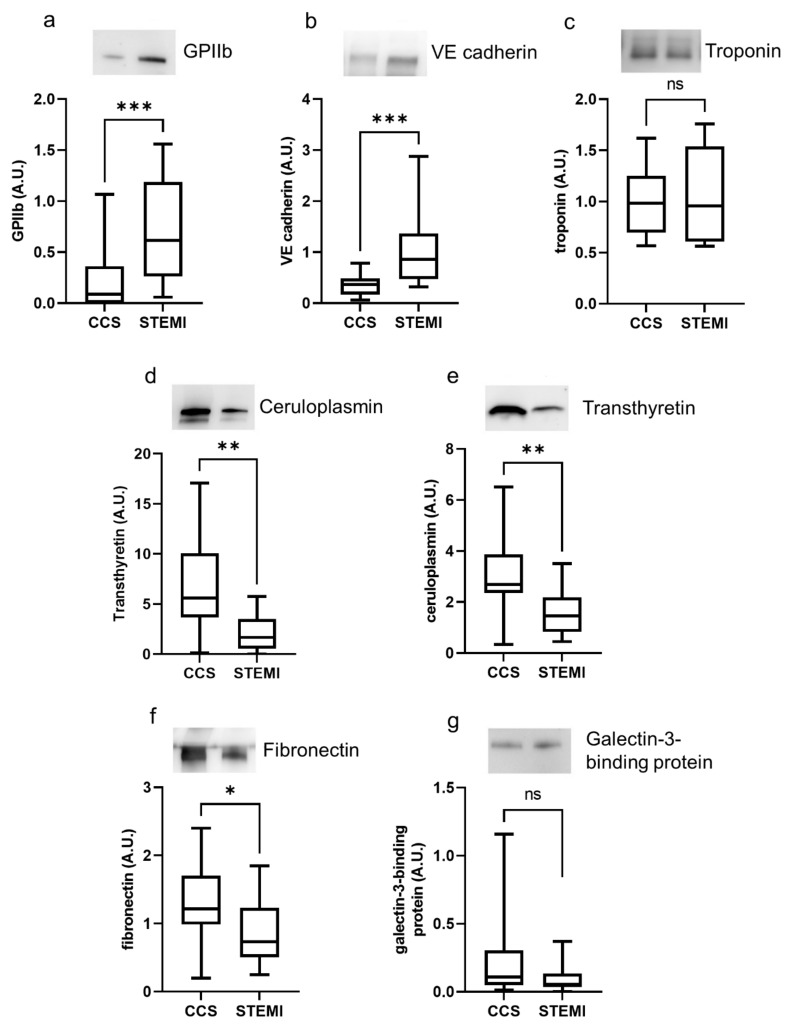
Plasma exosomes of STEMI patients display a different expression of specific proteins. Plasma exosomes from CCS and STEMI patients were lysed, and the expression of selected proteins was investigated. (**a**–**g**) Densitometric quantification and representative images of Western blot analysis of GPIIb (**a**), VE-cadherin (**b**), troponin (**c**), ceruloplasmin (**d**), transthyretin (**e**), fibronectin (**f**) and galectin-3-binding protein (**g**). For each box plot, the center line illustrates the median and box limits indicate the 25th and 75th percentiles. ns: not significant, * *p* < 0.05, ** *p* < 0.01, *** *p* < 0.001.

In STEMI patients, exosomes displayed a higher expression of platelet marker GPIIb and vascular endothelial marker VE-cadherin, but a similar amount of cardiac troponin, when compared to exosomes of CCS patients (Figure 2a–c). In addition, the acute-phase proteins ceruloplasmin, transthyretin and fibronectin were more expressed in exosomes of CCS patients than in those of STEMI patients (Figure 2d–f), whereas the expression of galectin-3-binding protein was similar in the two groups (Figure 2g). Similar behavior was observed when the total exosome protein cargo per mL of plasma was considered (Supplementary Materials Table S1).

### 2.3. Exosome Characterization in Uncomplicated STEMI versus OHCA-STEMI

**Figure 3:**
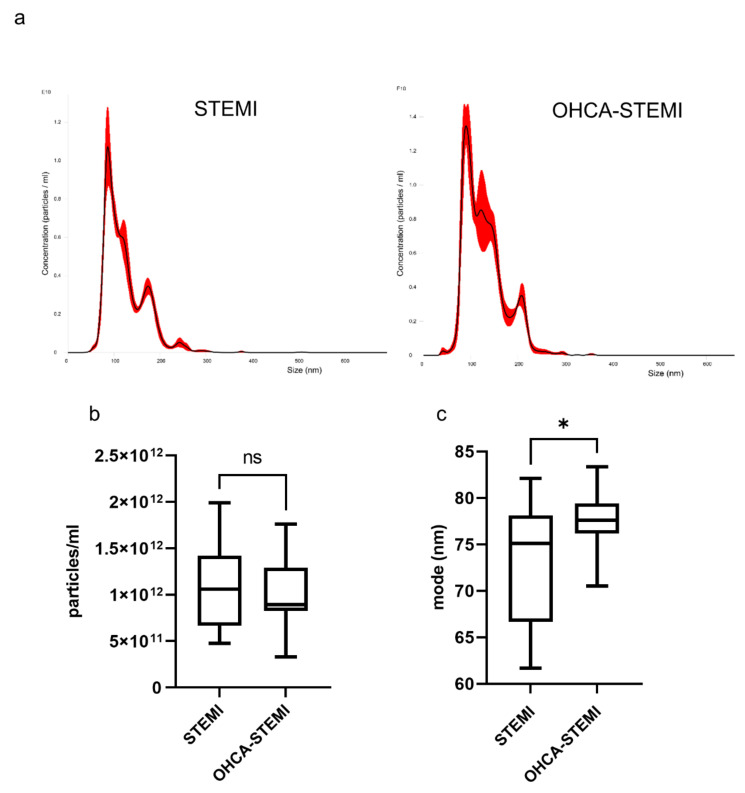
Exosomes isolated from plasma of STEMI with out-of-hospital cardiac arrest (OHCA-STEMI) have a higher dimension compared to uncomplicated STEMI. (**a**) Representative NTA traces of exosomes isolated from plasma of uncomplicated STEMI (*n* = 18) and OHCA-STEMI (*n* = 17). (**b**,**c**) Analysis of exosome concentration (number of particles/mL) (**b**) and dimension (mode) (**c**) as assessed by NTA. For each box plot, the center line illustrates the median and box limits indicate the 25th and 75th percentiles. ns: not significant, * *p* < 0.05.

Exosomes isolated from plasma of OHCA-STEMI were larger than those of uncomplicated STEMI, whereas exosome concentration was similar in the two STEMI groups (Figure 3a–c).

**Figure 4:**
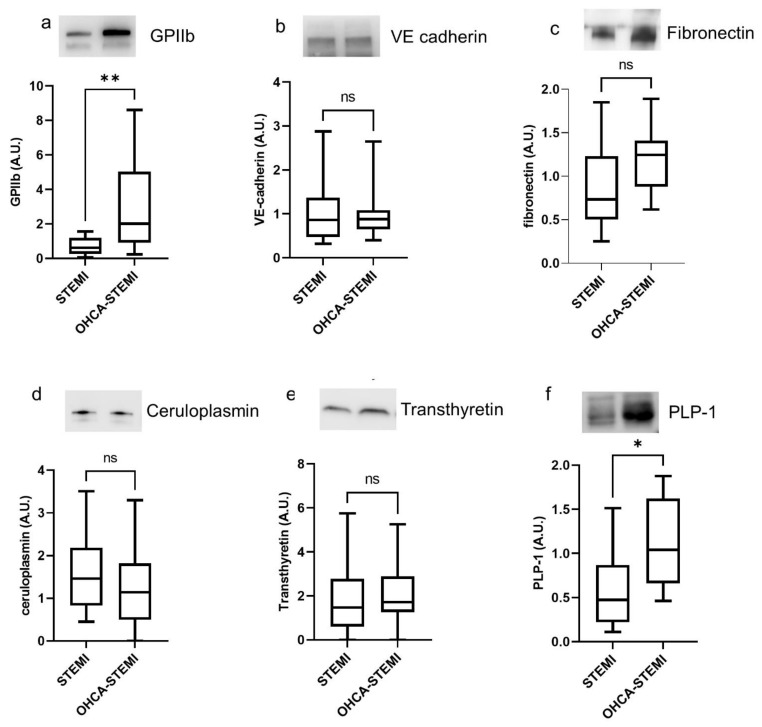
Plasma exosomes of OHCA-STEMI patients display a greater expression of platelet and brain-associated markers. (**a**–**f**) Densitometric quantification and representative images of Western blot analysis of GPIIb (**a**), VE-cadherin (**b**), fibronectin (**c**), ceruloplasmin (**d**), transthyretin (**e**) and PLP1 (**f**). For each box plot, the center line illustrates the median and box limits indicate the 25th and 75th percentiles. ns: not significant, * *p* < 0.05, ** *p* < 0.01.

Concerning the proteins previously shown to be differently expressed between STEMI and CCS patients (Figure 2), we found that the level of GPIIb was higher in exosome of OHCA-STEMI than in uncomplicated STEMI, whereas no difference in terms of fibronectin, VE-cadherin, ceruloplasmin and transthyretin expression was observed between the two STEMI groups (Figure 4a–e).

Finally, a greater amount of the brain-associated marker PLP1 was detected in exosomes isolated from OHCA-STEMI compared to uncomplicated STEMI (Figure 4f). Similar behavior was observed when the total exosome protein cargo per mL was considered (Supplementary Materials Table S2).

### 2.4. Exosome Ability to Discriminate between STEMI and CCS

**Figure 5:**
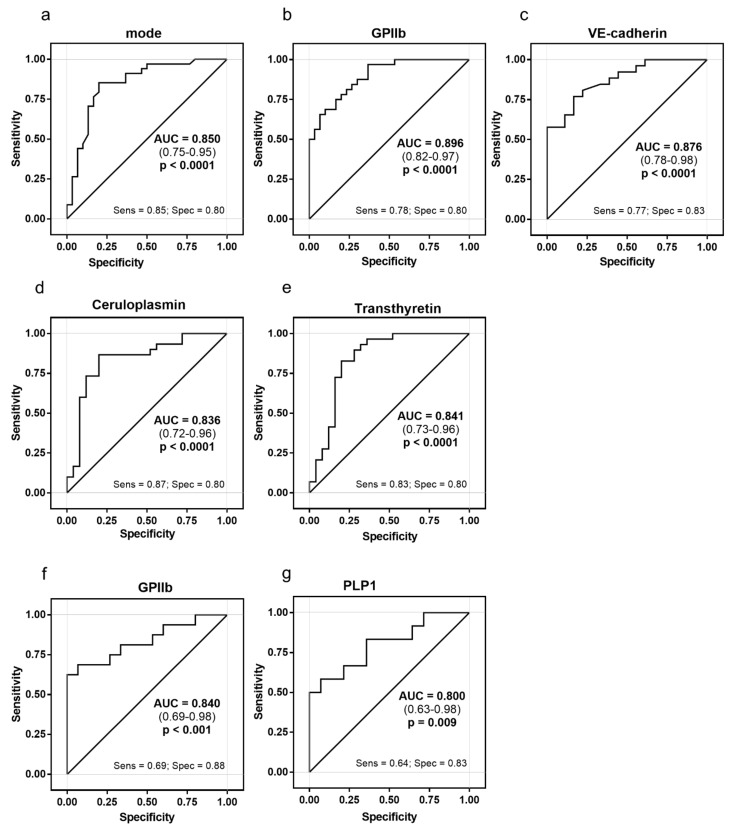
ROC curve analysis of exosomes versus cardiovascular clinical manifestation. Receiver operating characteristic (ROC) curve analysis was used to evaluate the ability of exosome to discriminate between STEMI (overall) and CCS patients (**a**–**e**) and between OHCA-STEMI and uncomplicated STEMI (**f**,**g**) in terms of dimension (mode) (**a**), GPIIb (**b**–**f**), VE-cadherin (**c**), ceruloplasmin (**d**), transthyretin (**e**) and/or PLP1 (**g**). Areas under the curve (AUCs), *p* values for AUC differences, cut-off values, sensitivity, and specificity are reported.

When the two STEMI groups were pooled together and compared with CCS patients, the exosome mode and the expression of GPIIb, VE-cadherin, ceruloplasmin and transthyretin accurately discriminated between STEMI and CCS patients, with the AUC ranging from 0.84 to 0.90 (Figure 5a–e and Supplementary Materials Table S3). In addition, the mode and the expression of GPIIb and PLP1 accurately discriminated (AUC 0.84 and AUC 0.80, respectively) between OHCA-STEMI and uncomplicated STEMI (Figure 5f,g and Supplementary Materials Table S3)

## 3. Discussion

In the present study, we show that circulating exosomes from STEMI patients have a different signature, in terms of number, dimension and specific protein cargo, compared to that of CCS patients. In addition, we observed that exosome expression of GPIIb and PLP1, specific proteins of platelets and brain cells, respectively, well discriminate OHCA-STEMI and uncomplicated STEMI, suggesting for these proteins a potential role as markers of brain injury during STEMI. 

The release of exosomes in the blood is known to be affected by disease conditions [30], including hypoxia [10,31]. In particular, exosomes have recently gained interest for their role in intercellular communication, representing a source of potentially critical information on the metabolic status of the cells and organs involved in the pathological event. Nevertheless, the exosome features and protein profile, as well as their biological meaning during STEMI, a critical condition characterized by acute coronary occlusion, are still unknown. Focusing on exosome content in this clinical setting, microRNAs are the most studied components [5,16,17]. To the best of our knowledge, no study so far has investigated the exosome protein cargo, as well as their cellular origin, in STEMI patients. Indeed, the literature available on protein profile in this topic is currently limited to extracellular vesicles studied indiscriminately as a pool collection of both microvesicles and exosomes [32,33]. Recently, Carrozzo and colleagues showed a different protein expression profile of plasma exosomes in older cardiac surgery patients before and after CABG or heart valve surgery (HSV) [9].

In our study, we found that the number and the size of circulating exosomes at hospital admission are significantly increased in STEMI patients as compared to CCS patients. This may represent an adaptive mechanism reflecting cellular hyperactivation and the increased cross-talk between cells and organs in response to the acute cardiac event [34].

Moreover, exosomes from STEMI patients have a 6-fold and 3-fold higher expression of GPIIb and VE-cadherin proteins, respectively, than CCS patients, mirroring the hyper-platelet activation and endothelial dysfunction related to this clinical event [35,36,37,38]. Conversely, no difference was observed in the exosome expression of troponin between the two study groups. This could be due to the fact that we isolated exosomes from plasma of STEMI patients in the very early hypoxic phase (at hospital admission), when thrombosis, endothelial damage and hypoxic/inflammatory pathways play a predominant role, whereas cardiomyocyte necrosis represents a later phenomenon. A delayed assessment of exosome protein cargo might have revealed significant differences also in troponin expression between STEMI and CCS patients. Moreover, among the acute-phase proteins investigated in our study, only transthyretin and ceruloplasmin discriminated between STEMI and CCS patients. These two proteins have been associated with cardiovascular diseases [22,39,40,41] and used as markers of inflammation. Indeed, during the acute-phase response/increased inflammation, the plasma level of ceruloplasmin increases, whereas the concentration of transthyretin drops [29,42,43,44,45].

In agreement with the association between low circulating levels of transthyretin and cardiovascular disease [39,40], we detected lower expression of transthyretin in exosomes of STEMI than in those of CCS patients. Conversely to the higher level of circulating ceruloplasmin found in myocardial infarction patients, exosomes isolated from STEMI displayed a reduced level of ceruloplasmin, suggesting that exosome protein expression does not necessarily reflect circulating free-form levels of this positive acute-phase protein [20,39]. Interestingly, changes in plasma exosome-associated inflammatory proteins, including ceruloplasmin, have been recently reported also in CABG and HSV patients [9].

Cardiac arrest, mainly due to ventricular fibrillation, is the most feared early complication in STEMI patients, associated with a 10-fold higher mortality rate [46]. Even if the number of STEMI patients with resuscitated cardiac arrest continues to increase, the majority of them have a worse prognosis and a variable degree of brain injury due to the transient oxygen deprivation occurring during cardiac arrest, followed by the organ reperfusion injury [47]. Hence, biomarkers providing early information on brain injury severity after cardiac arrest are of clinical relevance in order to improve prognostic stratification of the patients and to optimize therapeutic strategy. In this clinical scenario, exosomes may be potential indicators of brain injury detectable in the blood thanks to their ability to cross the blood–brain barrier [18,19,20,31]. Indeed, in vitro and in vivo studies showed that hypoxic, ischemic and traumatic brain injuries affect the release and the protein cargo of exosomes of cerebral origin [31,48,49,50,51,52] and their impact on brain performance. Exosomes released from oligodendrocytes may improve neuronal viability under cell stress conditions and promote neuronal survival during cerebral ischemia [53,54,55].

In line with this evidence, we found that exosomes isolated from OHCA-STEMI patients are bigger and display higher expression of oligodendrocyte PLP1 marker than those of uncomplicated STEMI, whereas no difference was observed in their total number. The significantly greater amount of brain PLP1 in circulating exosomes of OHCA-STEMI patients may reflect both the enhanced release of exosomes from suffering brain cells and/or the increased permeability of the blood–brain barrier occurring under hypoxic conditions [56].

Remarkably, we found the GPIIb was significantly more expressed in exosomes from OHCA-STEMI patients, which is in line with the knowledge of the hyperactivation of platelets observed during cerebral ischemia [57,58] and in patients with resuscitated cardiac arrest [59,60]. Although still preliminary, our findings suggest that in STEMI patients, the exosome profile characterizes those patients who experience brain suffering due to cardiac arrest. Future studies are needed to confirm our data and to investigate the potential clinical utility of exosome expression of GPIIb and PLP1 in reflecting brain injury extent and prognosis.

Our study must be considered in light of some limitations. First, we evaluated a small study population admitted to a single center. Therefore, no inference on the clinical relevance of our observations can be made. Second, our data generate hypotheses only since they do not provide evidence to support direct mechanisms underlying the connection between the exosome profile and patient’s clinical presentation. Experimental data are still required in suitably designed models to explore the specific mechanism(s) underlying the exosome profile, as well as its biological meaning. The differences in protein expression observed in our study should be confirmed in a larger patient population by using more manageable, sensitive and quantitative assays than Western blotting. Moreover, the information on neurological damage (e.g., complete neurological evaluation and neuro-MRI analyses) that would allow the clinical relevance of exosome protein cargo to be well appreciated is missing. We did not serially evaluate plasma exosomes in our STEMI cohort. As all STEMI patients underwent primary percutaneous coronary revascularization, we cannot exclude that exosome characterization performed after the procedure could have enhanced differences between study groups by incorporating the effect of reperfusion injury on the exosome. Finally, the impact of severity and extent of coronary atherosclerosis on exosome protein cargo has not been investigated and represents the topic of future investigations, based on larger study populations.

## 4. Materials and Methods

### 4.1. Study Population

This prospective study was conducted at the Centro Cardiologico Monzino, Milan, Italy. Consecutive male patients who were admitted to the Intensive Cardiac Care Unit for first STEMI without hemodynamic instability or other major clinical complications before hospital presentation (Killip class I) and who underwent primary percutaneous coronary intervention (pPCI) between September 2017 and October 2018 were considered. Moreover, consecutive male STEMI patients experiencing resuscitated out-of-hospital cardiac arrest (OHCA-STEMI) due to ventricular fibrillation and undergoing pPCI between September 2017 and December 2019 were also included in the study. Patients underwent pPCI if they had typical chest pain initiated within 12 h and at least 1 mm ST-segment elevation in two or more contiguous leads. Finally, consecutive male patients with CCS and with documented obstructive atherosclerotic disease at coronary angiography, performed between September 2017 and September 2018, were included in the study as controls. We excluded patients with chronic heart failure, valvular heart disease, acute and chronic infections, liver or renal disease, anemia, cancer, immunologic disorders and recent (<3 months) surgical procedures or trauma. The study was approved by the Ethical Committee of IRCCS Istituto Europeo di Oncologia and Centro Cardiologico Monzino (R726-CCM764), and written informed consent was obtained from all participants. This investigation conformed to the principles outlined in the Declaration of Helsinki.

### 4.2. Study Protocol 

Demographic, clinical, biochemical and echocardiographic data were obtained in all patients. In all STEMI patients, peripheral venous blood was drawn at hospital admission; in CCS patients, it was drawn before elective coronary angiography. We focused on specific proteins representing potential biosignature of acute myocardial injury (cardiac troponin), platelet activation/aggregation (glycoprotein IIb (GPIIb)), endothelial dysfunction (VE-cadherin), inflammation (transthyretin, ceruloplasmin, fibronectin, and galectin-3-binding protein) and brain injury (myelin proteolipid protein (PLP1)). This protein profile was compared between uncomplicated STEMI and CCS patients (control group) and, then, between uncomplicated STEMI patients and OHCA-STEMI.

### 4.3. Human Plasma Collection and Exosome Isolation 

Blood was drawn into EDTA tubes and processed to obtain plasma through centrifugation at 1800× *g* for 15 min at room temperature not later than 30 min after withdrawal. The collected plasma samples were stored at −80° until use. Exosomes were isolated from plasma using Invitrogen Total Exosome Isolation Kit (from plasma) (Thermo Fisher Scientific, Waltham, MA, USA) following the manufacturer’s instructions. Briefly, 250 µL of plasma was centrifuged at 10,000× *g* for 20 min at room temperature. The clarified plasma was diluted with phosphate-buffered saline (PBS) and the Exosome Precipitation Reagent was added. After mixing the sample thoroughly by vortexing, it was incubated at room temperature for 10 min and finally centrifuged at 10,000× *g* for 5 min at room temperature. The supernatant was carefully discarded and the pellet of exosomes was resuspended in PBS. The quality of exosomes isolated from plasma was thoroughly tested by different techniques (Supplementary Materials Figure S1), as strongly suggested by guidelines of the International Society for Extracellular Vesicles (ISEV) [61].

### 4.4. Nanoparticle Tracking Analysis (NTA)

Concentration and size distribution of particles in exosome samples were measured with NanoSight (NS300) (Malvern Panalytical Ltd., Malvern, UK) equipped with NTA software (version 3.4; Malvern Panalytical Ltd., Malvern, UK). All samples were diluted to the appropriate concentration, and five videos of 60 s were recorded for each sample and analyzed under constant settings using NTA software (version 3.4; Malvern Panalytical Ltd., Malvern, UK). The settings were established according to the manufacturer’s software manual (NanoSight NS300 User Manual, MAN0541-01-EN-00, 2017).

### 4.5. Western Blot Analysis

Exosomes were lysed using RIPA Buffer (Cell Signaling Technologies, Inc., Danvers, MA, USA), and a volume corresponding to 3 × 10^9^ particles was loaded onto a 4–20% polyacrylamide gel under reducing conditions; resolved proteins were transferred to PVDF Transferring Membranes of 0.2 µm pore size [62]. After blocking with 5% bovine serum albumin in PBS and 0.05% Tween 20, membranes were incubated overnight at 4 °C with the following primary antibodies: CD9 (NBP2-22187), TSG101 (NBP1-80659), GPIIb (NBP1-84581) and calnexin (NB100-1965SS) from Novus Biologicals (Centennial, CO, USA); transthyretin (11891-1-AP) and ceruloplamin (66156-1-lg) from Proteintech Group, Inc.; galectin-3-binding protein (VMA00292) from Bio-Rad Laboratories S.r.l.; fibronectin, kindly provided by Prof. L. Visai and Dr. F. Bertoglio and produced as previously described [30]; cardiac troponin (WH0007137M4) from Sigma-Aldrich; and PLP1 (MA1-80034) from Thermo Fisher Scientific. Antibody binding was assessed by horseradish peroxidase conjugated secondary antibodies, and immunoreactive bands were detected with Clarity Western ECL Substrate (Bio-Rad Laboratories S.r.l.) and were visualized on the Bio-Rad ChemiDoc XRS Imager system (Bio-Rad Laboratories S.r.l.). Quantification of band intensity was performed with Image Lab Software, version 6.0.1 (Bio-Rad Laboratories S.r.l.). Total exosome protein cargo per mL reported in Supplementary Materials Tables S1 and S2 was obtained as the product between AU protein expression and the number of exosomes in 1 mL.

### 4.6. Statistical Analysis

Statistical analysis was performed using the GraphPad Prism 8.0 Software (GraphPad Software Inc., San Diego, CA, USA) and SAS statistical software (v.9.4). The distribution of continuous variables was assessed by visual inspection of frequency histograms and with the use of the Shapiro–Wilk test. Continuous variables were expressed as mean ± standard deviation (SD) or median with interquartile range if they followed a normal or skewed distribution, respectively. Normal continuous variables were compared using the *t*-test for independent samples, variables not normally distributed were compared with the Wilcoxon rank-sum test and categorical data were compared using the chi-square test or the Fisher exact test. One-way ANCOVA with Dunnett correction for multiple comparisons was used to find clinical differences in the comparison of CCS or OCHA-STEMI with STEMI. The ability of individual exosome signatures to discriminate between STEMI overall and CCS or OHCA-STEMI and uncomplicated STEMI was assessed by ROC curve analyses. All performed analyses were adjusted for age. A *p* value <0.05 was considered to be statistically significant.

## 5. Conclusions

Here, we show, for the first time, that the exosome profile of STEMI patients has features different from that of CCS patients, which might likely reflect the pathophysiological mechanisms involved in STEMI. Further, the exosome expression of brain- and platelet-specific markers discriminate the patients with ischemic brain injury. Specific studies investigating the direct association between exosome protein cargo and severity of neurological damage will be crucial to their potential use as a clinical biomarker. Whether our preliminary observations pave the way to new biomarker discovery with potential therapeutic implications requires further investigation.

## Figures and Tables

**Table 1:** Clinical and laboratory data of the study population.

Variable	CCS (*n* = 32)	STEMI (*n* = 18)	OHCA-STEMI (*n* = 17)	P-ANOVA	* *p* Value *CCS* vs. *STEMI*	* *p* Value *STEMI* vs. *OHCA-STEMI*
**Demographic and clinical characteristics**						
Age, years	64.1 ± 8.1	67.2 ± 10.8	58.5 ± 10.5	0.03	0.42	0.02
BMI, Kg/m^2^	26.0 ± 3.0	26.5 ± 3.3	27.1 ± 4.4	0.71	0.70	1.00
RBC, 10^6^/μL	4.8 ± 0.4	4.0 ± 0.9	4.5 ± 0.8	0.0003	0.0001	0.03
WBC, 10^3^/μL	7.7 ± 1.9	11.6 ± 1.9	13.0 ± 4.1	<0.0001	<0.0001	0.37
PLT, 10^3^/μL	236.6 ± 44.3	242.4 ± 53.7	247.1 ± 61.3	0.053	0.08	1.00
Hemoglobin (g/dL)	14.6 ± 1.2	13.3 ± 2.0	14.2 ± 1.8	0.01	0.007	0.08
Total cholesterol, mg/dL	159.9 ± 30.4	172.4 ± 42.9	171.4 ± 43.2	0.32	0.22	0.55
HDL cholesterol, mg/dL	46.4 ± 10.4	41.5 ± 8.8	39.7 ± 9.4	0.10	0.12	1.00
LDL cholesterol, mg/dL	90.4 ± 28.6	106.1 ± 39.2	113.7 ± 31.9	0.08	0.09	0.98
Triglycerides, mmol/L	71.0 ± 63.4	128.8 ± 60.6	136.6 ± 72.8	0.29	0.26	0.96
Glycemia, mg/dL	109.4 ± 15.6	181.9 ± 102.3	206.8 ± 122.5	<0.0001	0.001	0.30
Creatinine, mg/dL	1.5 ± 0.2	1.1 ± 0.3	1.1 ± 0.3	0.18	0.99	0.18
CRP, mg/L	0.7 (0.5–2.3)	2.8 (1.7–3.8)	2.2 (0.9;4.4)	0.04	0.03	0.59
hs Tnl max, μg/L	/	16.3 (12.9–99.6)	80.3 (35.6–1742.7)	0.26	/	0.26
Time-to-Pres (h)	/	3.36 ± 2.2	5.24 ± 7.38	0.33	/	0.33
anterior AMI, *n* (%)	/	10 (55.6%)	10 (58.8%)	0.78	/	0.78
Smokers, *n* (%)	6 (71.9%)	7 (38.9%)	6 (35.3%)	0.84	0.98	0.79
Diabetes, *n* (%)	6 (19.4%)	6 (33.3%)	5 (29.4%)	0.54	0.51	0.99
Hypertension, *n* (%)	23 (71.9%)	12 (66.7%)	9 (52.9%)	0.66	0.66	1.00
Familiarity, *n* (%)	4 (22.2%)	4 (22.2%)	2 (11.8%)	0.01	0.11	0.43
Dyslipidemia, *n* (%)	20 (62.5%)	9 (50%)	5 (29.4%)	0.15	0.49	0.62
**Pharmacological treatments**						
ACE inhibitors, *n* (%)	21 (67.7%)	5 (27.8%)	8 (47.1%)	0.02	0.008	0.20
Statin, *n* (%)	23 (74.2%)	2 (11.1%)	3 (17.6%)	<0.0001	<0.0001	0.74
β-blockers, *n* (%)	19 (61.3%)	4 (22.2%)	4 (23.5%)	0.006	0.007	0.83
Ca channel blockers, *n* (%)	5 (16.1%)	1 (5.6%)	1 (5.9%)	0.39	0.46	0.99
Hypoglycemics, *n* (%)	4 (12.9%)	5 (27.8%)	2 (11.8%)	0.44	0.36	0.49
Thienopyridine, *n* (%)	11 (35.5%)	0 (0%)	1 (5.9%)	0.002	0.003	0.76
Aspirin, *n* (%)	27 (87.1%)	3 (16.7%)	3 (17.6%)	<0.0001	<0.0001	0.78

BMI: body mass index, RBC: red blood cells; WBC: white blood cells; PLT: platelet; HDL: high-density lipoprotein; LDL: low-density lipoprotein; CRP: C-reactive protein; hs Tnl: high-sensitivity troponin; ACE: angiotensin-converting enzyme; CCS: chronic coronary syndrome; AMI: acute myocardial infarction; STEMI: ST-segment elevation myocardial infarction; OHCA-STEMI: STEMI experiencing resuscitated out-of-hospital cardiac arrest. * age-adjusted *p* value with Dunnett correction for multiple comparisons.

## Data Availability

Not applicable.

## Supplementary Materials

The following are available online at https://www.mdpi.com/article/10.3390/ijms22158065/s1, Figure S1: Characterization of extracellular vesicles isolated from plasma of patients, Table S1: Total exosome protein cargo per mL in CCS and uncomplicated STEMI patients, Table S2: Total exosome protein cargo per mL in uncomplicated STEMI and OHCA-STEMI patients, Table S3: Relation of plasma exosome profile in STEMI and OHCA-STEMI patients
