# Supplementary material for: Plasma Exosome Profile in ST-Elevation Myocardial Infarction Patients with and without Out-of-Hospital Cardiac Arrest
Source: Int J Mol Sci. 2021 Jul 28;22(15):8065. doi: 10.3390/ijms22158065 (PMC8347807; doi:10.3390/ijms22158065)
Supplement: Supplementary file 1 [file ijms-22-08065-s001.zip › ijms-1298948-supplementary.pdf]

Plasma exosome profile in ST-elevation myocardial infarction patients with and without out-of-hospital cardiac arrest.

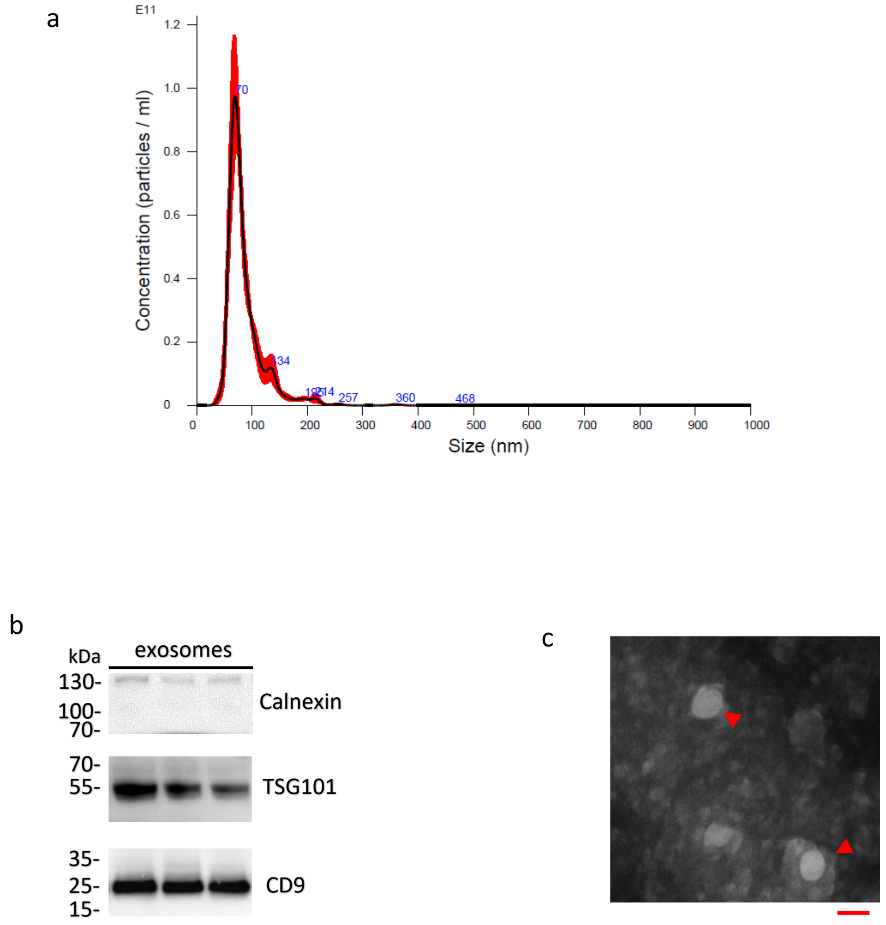

Figure S1: Characterization of extracellular vesicles isolated from plasma of patients. The quality of vesicles isolated from plasma of patients was carefully checked by different techniques, as strongly suggested by guidelines of the International Society of the Extracellular Vesicles (ISEV). (**a**) Representative NTA profile showing a major peak of abundant vesicles ranging from around 50 nm to 130 nm, thus the expected exosome dimension. (**b**) Western blot analysis revealed the presence of largely recognized positive exosomal markers (CD9 and TSG101) whereas endoplasmic reticulum-localized protein calnexin was not detected. (c) Representative TEM image confirming the presence of intact vesicles (scale bar:100 nm).

Table S1: Total exosome protein cargo per ml in CCS and uncomplicated STEMI patients

Protein (A.U./ml)	CCS	STEMI	P value
GPIIb	0 (0-77.41)	210 (97.36-298.3)	**<0.0001**
VE-Cadherin	103.1 (44.84-156.1)	243.3 (177.3-522.6)	**<0.0001**
Troponin	295 (235.2-588.7)	401.4 (267.6-708.9)	0.322
Ceruloplasmin	663.8 (474.4-950.2)	459.8 (342.1-679.3)	**0.016**
Fibronectin	326.2 (236.4-412.8)	240.6 (171.3-324.7)	**0.036**
Transthyretin	1613 (912.7-2459)	461 (274.6-884.7)	**0.0004**
Galectin-3-binding protein	30.12 (13.94-79.77)	24.53 (12.86-40.39)	0.476

A.U.: Arbitrary Unit; GPIIb: Glycoprotein IIb

Table S2: Total exosome protein cargo per ml in uncomplicated STEMI and OHCA-STEMI patients

Protein (A.U./ml)	STEMI	OHCA-STEMI	P value
GPIIb	210 (97.36-298.3)	573.6 (257.6-1598)	**<0.0001**
VE-Cadherin	243.3 (177.3-522.6)	252.9 (235.1-365.5)	0.801
Ceruloplasmin	459.8 (342.1-679.3)	415.6 (148.2-580.7)	0.394
Fibronectin	240.6 (171.3-324.7)	362.1 (315.3-501.4)	0.0510
Transthyretin	461 (274.6-884.7)	503.1 (326.3-788.1)	**>0.999**
PLP1	167.5 (79.33-303.3)	352 (239.5-530.5)	**0.009**

A.U.: Arbitrary Unit; GPIIb: Glycoprotein IIb; PLP1: Myelin proteolipid protein

Table S3: Relation of plasma exosome profile in STEMI and OHCA-STEMI patients

CCS vs STEMI (overall)	AUC	95% Confidence Interval	P value
concentration	0.692	0.563-0.820	0.007
mode	0.849	0.750-0.948	**<0.0001**
GPIIb	0.896	0.822-0.970	**<0.0001**
VE-Cadherin	0.876	0.778-0.975	**<0.0001**
Ceruloplasmin	0.836	0.722-0.950	**<0.0001**
Transthyretin	0.841	0.725-0.958	**<0.0001**
Fibronectin	0.647	0.492-0.803	0.075
**STEMI vs OHCA-STEMI**			
concentration	0.565	0.369-0.762	0.509
mode	0.700	0.523-0.876	**0.047**
GPIIb	0.838	0.694-0.981	**0.001**
VE-Cadherin	0.503	0.269-0.737	0.979
Ceruloplasmin	0.616	0.412-0.820	0.280
Transthyretin	0.603	0.386-0.821	0.345
Fibronectin	0.744	0.557-0.932	**0.034**
PLP1	0.804	0.631-0.976	**0.009**

GPIIb: Glycoprotein IIb; PLP1: Myelin proteolipid protein
